# Isolated Abducens Nerve Palsy After Herpes Zoster Ophthalmicus: A Case Report

**DOI:** 10.22336/rjo.2023.65

**Published:** 2023

**Authors:** Rizaldy Taslim Pinzon, Marlyna Afifudin, Isa Karuniawati

**Affiliations:** * Duta Wacana Christian University, School of Medicine, Yogyakarta, Indonesia

**Keywords:** herpes zoster ophthalmicus, varicella-zoster virus, cranial nerve VI palsy

## Abstract

**Aim:** The purpose of this study was to demonstrate a case of herpes zoster in the patient.

**Methods:** Case report.

**Results:** Herpes zoster ophthalmicus is a rare but well-known cause of CN VI palsy that affects an elderly patient due to a reduction in the immunity to the Varicella Zoster Virus (VZV). We reported a case of herpes zoster in our patient, a 67-year-old Javanese female who presented with a VI nerve palsy within 1 week after the vesicular rash. Our patient received Valacyclovir, Gabapentin, and steroid treatment, then responded quite well to the combination of these therapies without side effects as the goals were to diminish acute and chronic pain, fasten the healing of the skin and nerve, and reduce the chances of dissemination. Based on studies, systemic antivirals should be given in all cases of HZO to minimize complications and steroids should not be given without antiviral therapy so as not to increase viral replication.

**Conclusions:** As a complication of HZO, ophthalmoplegia may have various origins. We reported a case of sixth nerve palsy in HZO.

**Abbreviations:** HZO = herpes zoster ophthalmicus, VZV = varicella-zoster virus, CN = Cranial Nerve

## Introduction

Herpes zoster ophthalmicus (HZO) is a rare condition when varicella-zoster virus (VZV) replicates and produces facial lesions in areas supplied by sensory branches of the ophthalmic division of the fifth nerve [**1**]. Herpes zoster ophthalmicus (HZO) is the involvement of the ophthalmic division (V1) of the trigeminal nerve and appears in 10–20% of all cases of herpes zoster [**1**,**2**]. The HZO can also cause a variety of ocular complications. The acute course of HZO is usually benign. Some serious ocular complications have been documented. Extraocular muscle involvement is a rare complication occurring in only seven to 31% of HZO [**1**,**2**]. The weakness is usually seen in elderly patients and can be a self-limited resolution in most cases [**2**,**3**]. We reported a case of sixth nerve palsy that presented within a week of the onset of a zosteriform rash.

## Case report

**Fig. 1:**
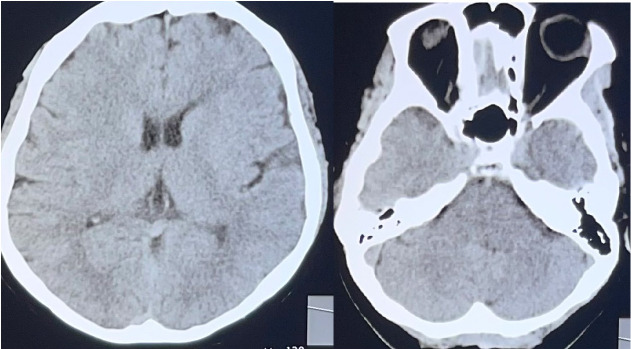
The brain CT was normal

A 67-year-old Javanese female presented to our Emergency Department with complaints of severe right-sided headache. The patient was fully alert, with no sign of weakness, cranial nerve examination was normal. No sign of neck rigidity and meningeal sign. Both deep tendon reflexes were normal, and no sign of pathological reflex was present. The headache had a sudden onset, with a pulsating and burning sensation. No vomiting or nausea, and no photophobia were present. The brain CT scan was unremarkable (**Fig. 1**). The laboratory blood test was also unremarkable. No sign of abnormality in the blood test, lung X-ray, or ECG, diplopia, and limitation of abduction in the left eye was observed. Her medical history was unremarkable. Both systemic examination and blood pressure assessments were completely normal. The patient was treated with paracetamol infusion, followed by ketorolac injection. The pain only minimally diminished. 

**Fig. 2:**
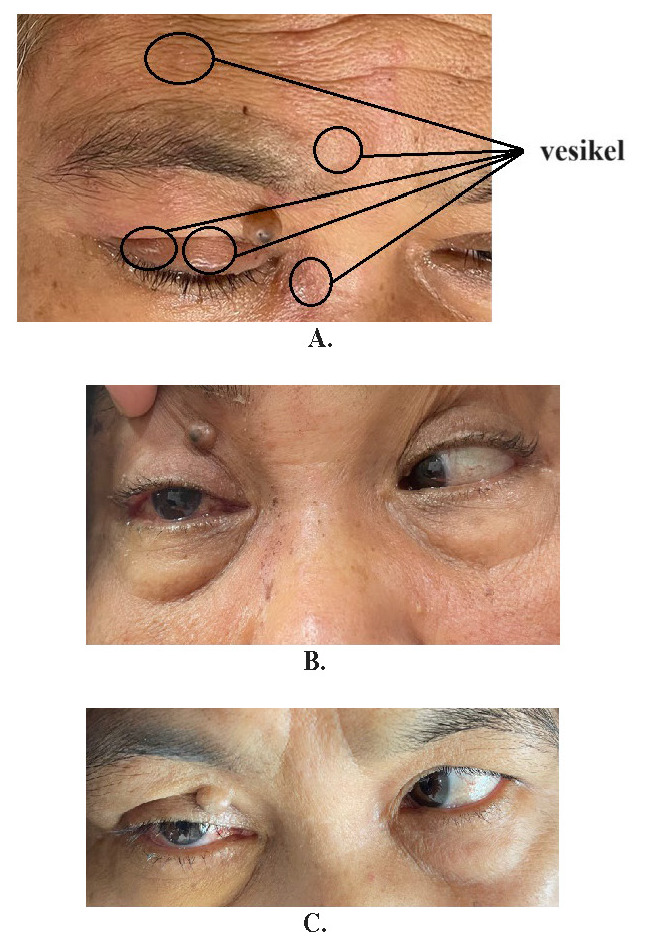
The beginning of the rash in the third days of hospitalization (**A**), the follow-up in two weeks (**B**), and the follow-up in 2 months (**C**)

After three days of hospitalization, she developed multiple painful vesiculobullous eruptions on the right side of her forehead (**Fig. 2 A**). No specific findings were present on the further neurological examination. Diagnostic workup, for an underlying primary or acquired immunodeficiency, turned out to be negative. Laboratory evaluation results, including erythrocyte sedimentation rate, were all within normal limits. Temporal arteries were non-palpable.

At our initial examination, the patient’s visual acuity was 20/20 in the right eye and 20/30 in the left eye. Extraocular muscle movements were painless with restricted abduction of the right eye, compatible with a right CN VI palsy (**Fig. 2 B**). Slit-lamp examination and fundoscopy showed mild-moderate conjunctival injection. The intraocular pressure was 14 mm Hg bilaterally.

A diagnosis of right cranial nerve VI palsy due to HZO was made, and the patient was started on oral valacyclovir 3,000 mg/day for a week. We administered steroid and gabapentin 3 times 100 mg daily. The pain was diminished slowly. The skin lesions resolved within 2 weeks and a gradual improvement in ocular motility was noted over the following months. In the 3rd month of follow-up, the patient presented with an incomplete recovery of CN VI function with a residual abduction deficit (**Fig. 2 C**). At that time, her vision was 20/20 in both eyes. During follow-up, the patient had no post-therapeutic neuralgia and suffered no major side effects due to the treatment.

## Discussion

Herpes zoster (HZ) is an uncommon disease mainly affecting elderly patients due to a reduction in immunity due to VZV [**2**]. It occurs by reactivation of latent virus in ganglions following primary infection with varicella zoster and appears mostly in thoracic and cranial sensory ganglia [**2**,**3**]. HZ is a localized disease characterized by unilateral radicular pain and a vesicular eruption. In addition to sensory neurons, HZ may also affect motor neurons in rare cases [**3**,**4**]. The ophthalmic division involvement of the trigeminal nerve is affected, the condition being referred to as HZO, which occurs in 10-25% of HZ, with 50% of these cases infecting the eye [**4**,**5**].

Ocular manifestations can be acute, chronic, and relapsing; and include conjunctivitis, keratitis (commonly epithelial followed by nummular and disciform), episcleritis, scleritis, uveitis, secondary glaucoma, cataract, and retinal necrosis [**4**]. HZO may cause extraocular muscle palsies of the third, fourth, and sixth cranial nerves in 7-31% of patients [**1**]. The third nerve is the most common site among them and the fourth the least [**5**,**6**]. Complete ophthalmoplegia is a rare manifestation of HZO, and only very few cases have been reported in the literature [**5**,**6**].

The extraocular muscle palsies usually appear 1-4 weeks after the rash but sometimes occur simultaneously with the rash or more than 4 weeks later [**2**,**3**]. Our patient presented with a sixth nerve palsy within 1 week after the vesicular rash. The pathogenesis of ophthalmoplegia is controversial and several mechanisms have been hypothesized: (1) a direct cytopathic effect from the virus itself on the surrounding neural tissue; (2) an immune response of the central nervous system to the virus; (3) occlusive vasculitis induced by the virus [**4**-**6**]. 

The therapeutic goal is to limit the severity of acute and chronic pain, fasten the healing of the skin and nerve, and reduce the chances of dissemination [**5**,**6**]. The treatment of HZO-related CN palsy is non-conclusive. There is an ongoing debate regarding the importance of systemic antiviral treatment and corticosteroids [**5**-**7**]. Systemic antivirals have been reported to reduce the risk of viral dissemination. Besides, their beneficial effects in reducing the clinical signs of HZO have been demonstrated. Therefore, we believe that antiviral therapy should be given in all HZO cases to minimize the frequency of potentially sight-threatening complications. Some authors have recommended systemic corticosteroids to treat the possible inflammatory component and/or to prevent postherpetic neuralgia [**6**-**8**]. It is well known that steroids should not be given alone (without antiviral therapy) due to concerns about the promotion of viral replication. Additionally, they may increase the risk of secondary skin infection [**5**-**7**]. In our case, the patient responded quite well to valacyclovir and steroid treatment and had no side effects. There are different suggestions for the treatment of paralytic lesions that complicate HZ. As the paralytic lesions tend to resolve spontaneously, the effects of specific treatments are unclear [**7**,**8**]. The prognosis of ophthalmoplegia is generally favorable. It was reported that the duration of diplopia associated with ocular motor palsy was between 2 and 23 months [**9**,**10**].

## Conclusion

As a complication of HZO, ophthalmoplegia may have various origins. We reported a case of sixth nerve palsy in HZO. 

**Conflict of Interest Statement**

The authors state no conflict of interest. 

**Informed Consent and Human and Animal Rights Statement**

Verbal informed consent has been obtained from the patient and family.

**Authorization for the use of human subjects**

Ethical approval: The research related to human use complies with all the relevant national regulations and institutional policies, as per the tenets of the Helsinki Declaration, and has been approved by the Ethics Committee of Duta Wacana Christian University, School of Medicine, Yogyakarta, Indonesia.

**Acknowledgments**

None.

**Sources of Funding**

None.

**Disclosures**

None.
